# CaMad2 Promotes Multiple Aspects of Genome Stability Beyond Its Direct Function in Chromosome Segregation

**DOI:** 10.3390/genes10121013

**Published:** 2019-12-05

**Authors:** Maicy L. Vossen, Hanaa M. Alhosawi, Katherine J. Aney, Laura S. Burrack

**Affiliations:** Department of Biology, Gustavus Adolphus College, Saint Peter, MN 56082, USA

**Keywords:** Mad2, spindle assembly checkpoint, genome instability, *Candida albicans*, fluconazole

## Abstract

Mad2 is a central component of the spindle assembly checkpoint required for accurate chromosome segregation. Additionally, in some organisms, Mad2 has roles in preventing mutations and recombination through the DNA damage response. In the fungal pathogen *Candida albicans*, CaMad2 has previously been shown to be required for accurate chromosome segregation, survival in high levels of hydrogen peroxide, and virulence in a mouse model of infection. In this work, we showed that CaMad2 promotes genome stability through its well-characterized role in promoting accurate chromosome segregation and through reducing smaller scale chromosome changes due to recombination and DNA damage repair. Deletion of *MAD2* decreased cell growth, increased marker loss rates, increased sensitivity to microtubule-destabilizing drugs, and increased sensitivity to DNA damage inducing treatments. CaMad2-GFP localized to dots, consistent with a role in kinetochore binding, and to the nuclear periphery, consistent with an additional role in DNA damage. Furthermore, deletion of *MAD2* increases growth on fluconazole, and fluconazole treatment elevates whole chromosome loss rates in the *mad2∆/∆* strain, suggesting that CaMad2 may be important for preventing fluconazole resistance via aneuploidy.

## 1. Introduction

Genome instability arises from many different causes, including mistakes in DNA replication, unrepaired DNA damage, and mis-segregation of chromosomes. These processes result in genetic variation among cells in a population. Typically, most mutations and aneuploidy events are detrimental to cells. However, in certain stressful circumstances, mutations and aneuploid chromosomes can have a selective advantage. High mutation rates are observed in human cancers and in several fungal pathogens, especially during the infection process. For example, large scale chromosomal changes rapidly generate genome diversity in *Candida albicans* following exposure to the mouse oral cavity [1]. Genome instability is also associated with antifungal drug resistance. In *C. albicans*, aneuploid cells with an isochromosome—two extra copies of 5L—have fluconazole resistance, and other specific aneuploid chromosomes can provide cross-resistance to multiple chemotherapeutic and antifungal drugs [2,3]. Aneuploidy also drives fluconazole resistance in the fungal pathogen *Cryptococcus neoformans* [4]. Therefore, cells must balance genome maintenance with mechanisms that promote genetic diversity in order to thrive in favorable conditions and survive in stressful environments.

Genome maintenance depends on the function of the spindle assembly checkpoint (SAC). A major role of the SAC is to detect unattached kinetochores. At unattached chromosomes, the mitotic checkpoint complex (MCC) binds to the anaphase-promoting complex (APC/C) to prevent cells from dividing before attachments are made. In the complex, Mad2 binds Cdc20, and together with BUBR1 (Mad3 in yeasts), prevents APC/C activity. Once the chromosomes are correctly aligned and spindle fibers are attached to the kinetochore, the MCC is disassembled, allowing APC/C activity and progression into anaphase through degradation of securin [5]. In addition to its roles in chromosome segregation, the SAC has recently been shown to respond to DNA damage and replication stress. The DNA damage response kinases ATR and CHK1 have been functionally linked with SAC components, including Mad2. SAC components delay metaphase in the presence of DNA damage. In *Caenorhabditis elegans* germ cells, Mad2 relocalizes to the nuclear periphery in a DNA damage response-dependent manner [6]. Similarly, relocalization of SAC components in response to DNA damage occurs in human cells [6]. In budding yeast, Mad2 has also been shown to have additional roles outside of mitosis to control DNA synthesis and allow cell survival in response to replication stress [7].

Mad2 is a central component of the spindle assembly checkpoint and is required for inhibition of APC/C. Mad2 exists in two forms, open and closed. The closed form is the form that allows Mad2 complex formation in the MCC and occurs when Mad2 is bound to Mad1 or Cdc20 [5]. In interphase, Mad1–Mad2 complexes are present at the nuclear pore in humans [8], budding yeast [9], and the hyphal fungus *Aspergillus nidulans* [10]. As cells begin to divide, Mad2-GFP localizes to kinetochores in response to non-attached kinetochores/mitotic block [11], but Mad1 and Mad2 remain at the nuclear periphery through mitosis in budding yeast when kinetochores are attached properly [9,12].

Mad2 binding to the kinetochore in yeast depends on two kinases, Bub1 and Mps1^Mph1^. Mps1^Mph1^ kinase activity is required for assembly of the Mad1–Mad2 core complex [5]. Bub1 is an essential SAC kinase and is required to recruit other SAC proteins [5]. In humans, the MCC is recruited by two separable pathways, KNL1-Bub3-Bub1 and Rod-Zwilch-Zw10 (RZZ). Recent evidence shows that efficient mitotic checkpoint signaling in human cells depends on the activity of both Bub1 and the RZZ complex [13]. However, yeast lack RZZ and require Bub1 for detection of unattached kinetochores. Bub1 binds to the kinetochore in connection with the KNL1^Spc105/Spc7^ kinetochore protein phosphorylated by Mps1^Mph1^ and Bub3. Heterodimers of Mps1^Mph1^ and Bub1 together with Mad1, Mad2 and Mad3 are sufficient to trigger metaphase arrest in both budding and fission yeast [14]. 

Overall, the SAC is well-conserved in diverse organisms. However, differences in functions between have been characterized, and some lineages, such as species in the bipolar budding yeast genus *Hanseniaspora*, have lost numerous genes in the SAC, including *MAD1* and *MAD2* [15]. *C. albicans* has many conserved functions and processes in common with other well-characterized yeasts. However, there are also several important differences in the genome dynamics of *C. albicans.* One notable difference is the parasexual process for recombination-based genetic diversity (rather than meiosis) [16]. Another difference is the presence of an H2A variant that cannot be phosphorylated by the Bub1 checkpoint protein [17]. Additionally, CaMps1 has roles in SAC activation but also has roles in morphological transitions from yeast to hyphal forms and in the oxidative stress response [18]. In *C. albicans*, CaMad2 has previously been shown to be important for accurate chromosome segregation, survival in high levels of hydrogen peroxide, and virulence in a mouse model of infection [19]. However, questions about the roles of CaMad2 in preventing sub-chromosomal genome instability and in evolving drug resistance remain unanswered.

In this work, we studied the roles of CaMad2 to learn more about the evolutionary conservation of the function of the protein in function and to better characterize the role of CaMad2 in promoting genome stability in the fungal pathogen, *C. albicans*. We found that CaMad2 promotes genome stability through its well-characterized role in promoting accurate chromosome segregation and through reducing smaller scale chromosome changes due to recombination and DNA damage repair. Additionally, the most commonly observed localization pattern of CaMad2-GFP at the nuclear periphery is consistent with a role in DNA damage response. Together, these results indicate that the DNA damage response through Mad2 is conserved in *C. albicans*. Interestingly, we also found that similar to other strains with altered chromosome segregation checkpoint function, deletion of *MAD2* increases growth on fluconazole, suggesting that disruption of any of these genes may promote the evolution of drug resistance in *C. albicans*. 

## 2. Materials and Methods 

### 2.1. Strain Construction

*C. albicans* strains are listed in Table S1. Lithium acetate transformation of linearized plasmids or PCR products with at least 70 bp of homology to the targeted gene were used for strain construction. Briefly, strains to be transformed were inoculated in YPA-glucose and grown at 30 °C for 16–18 h. Overnight cultures were then diluted 1:166 in YPA-glucose and grown at 30 °C for 3–4 h. Cells were washed with water, then TELiAc (10 mM Tris pH 7.5, 1 mM EDTA, 100 mM LiAc), and incubated in TELiAc with transformation DNA and 50 µg of sheared salmon sperm DNA for 30 min. Four volumes of PLATE mix (40% PEG, 10 mM Tris pH 7.5, 1 mM EDTA, 100 mM LiAc) were then added, and the transformation mix was incubated for 12–18 h at 20–24 °C. Transformations were heat-shocked at 42 °C for 1 h and then plated on selective media. Strains were checked by PCR of genomic DNA.

For the construction of the *MAD2* heterozygous and homozygous deletion strains, we replaced the *MAD2* gene with *URA3*, *HIS1*, and/or *ARG4* genes. We amplified the markers using primers with flanking 5’ and 3’ ends containing 70 bp homology to the targeted genes of interest from plasmids pGEM-*URA3,* pGEM-*HIS1,* and pRS-*ARG4*ΔSpe1 [20]. Correct insertions and the absence of native *MAD2* gene in homozygous deletion strains were verified with PCR. The *MAD2/MAD2-GFP* strain was tagged at the C-terminus with PCR-mediated transformation. Primers containing at least 70 bp homology to the targeted gene were used to amplify the GFP and marker from the C-terminal GFP tagging plasmids with HIS1 markers [21]. Fusion of GFP to the *MAD2* gene was confirmed by PCR. To construct the *PCK1p*-*CSE4* strains, the *URA3-PCK1p-CSE4* plasmid [22] was linearized with *EcoRV*. Integration into the *CSE4* upstream region was confirmed by PCR.

### 2.2. Growth Analysis

Strains were inoculated into 5 mL liquid YPA-glucose media and growth overnight at 30 °C with shaking. Overnight cultures were diluted into liquid media: YPA-glucose, YPA-succinate, YPA-glucose-nocodazole (50 µM), YPA-glucose-thiabendazole (222 µg/mL), YPA-glucose-hydrogen peroxide (1 mM), or YPA-glucose-fluconazole (1 µg/mL). Then, 150 μL of the 10^−3^ dilution was plated into a clear 96-well plate. Growth was determined by measuring the optical density of cultures (four cultures per condition) at 600 nm. Data was collected every 15 min using a Tecan Sunrise at 30 °C with shaking between cycles for 24 h. Data were analyzed using a custom Excel Macros program (a template version is included as File S1).

### 2.3. Serial Dilution and Plate Spotting

Strains were inoculated into 5 mL liquid YPA-glucose media and grown overnight at 30 °C with shaking. These strains were serially diluted using a deep 96 well plate from 10^−1^ to 10^−6^ in sterilized water. Dilutions were then spotted onto YPAD (yeast peptone adenine dextrose agar), YPAD-fluconazole (1 µg/mL), and YPAD-nocodazole (50 µM) agar plates. For UV light treatment, strains were exposed to 100 mJ/cm^2^ or 200 mJ/cm^2^ using a Stratalinker crosslinker (Stratagene, La Jolla, CA, USA). Plates were incubated at 30 °C for 24 h. Plates shown are representative of at least three biological replicates.

### 2.4. Minimum Inhibitory Concentration Assay

Strains were streaked for single colonies and grown on YPAD at 30 °C for 24 h. The inoculum was prepared by diluting twenty single colonies from each plate (~0.5 mm each) into 5 mL of sterile water, adjusting the sample to match a 0.5 McFarland standard, and then further diluting to 1:10 in sterile water. Fluconazole was diluted in two-fold from 128 µg/mL to 0.25 µg/mL 2× RPMI-MOPS pH 7, 2% glucose [23]. Uridine was added at a final concentration of 0.1 mg/mL for strains lacking *URA3.* 100 µL 2× media with fluconazole was combined with 100 µL of the inoculum for final fluconazole concentrations from 64 µg/mL to 0.125 µg/mL, and the plate was incubated at 37 °C for 24 h. Optical density at 600 nm was read with a Tecan Sunrise plate reader (Tecan, Männedorf, Switzerland). At least six biological replicates were tested for each strain.

### 2.5. Fluctuation Analyses of Chromosome Loss Rates

Fluctuation analysis of loss rates was performed similar to prior studies [24] using the method of the median [25]. Briefly, strains were streaked for single colonies and grown on YPAD for 2 days at 30 °C. Per strain, 8 independent colonies were inoculated into 500 μL liquid media (YPA-glucose, YPA-succinate, or YPA-glucose-fluconazole (1 µg/mL)), and grown overnight at 30 °C with shaking. Cultures were diluted with sterile water and plated onto nonselective YPAD for total cell counts and selective media (5-FOA for *URA3* loss). Plates were incubated at 30 °C for 1–3 days, and colony counts were used to calculate the rate of marker *URA3* loss per cell division.

### 2.6. SNP-RFLP 

At least 8 individual colonies that lost *URA3* were isolated from 5-FOA plates after incubation in the fluctuation analysis. Colonies were streaked on 5-FOA plates and incubated at 30 °C for 48 h. Following genomic DNA extraction, PCR was performed on the right (5R) and left (5L) ends of Chr5 using primers 10080A and 2340/2493; the right (3R) and left (3L) ends of Chr3 using primers 2091/2447 and 1765/2519; and the right (1R) and left (1L) ends of Chr1 using primers 2106/2441 and 1799/2450. Restriction digests were performed on resulting PCR products with AluI and Taq1 for Chr5, DdeI and Hpy188I for Chr3, and AluI and PstI for Chr1. Restriction digests were incubated at 37 °C (AluI, DdeI, Hyp188I, and PstI) or 60 °C (Taq1) for 8 h. Restriction digests were run on 3% agarose gels to check for SNP homozygosity or heterozygosity (digested or non-digested alleles based on SNP present within PCR) [26].

### 2.7. Reverse Transcriptase qPCR

Strains were inoculated into YPA-glucose and grown at 30 °C for 16–18 h. Cultures were then diluted 1:100 into YPA-glucose and grown at 30 °C for 4 h. RNA was prepared using the MasterPure yeast RNA purification kit (Epicentre, Madison, WI, USA) according to the manufacturer’s instructions. RNA was treated with DNase (Epicentre) to remove contaminating genomic DNA. cDNA was prepared using the ProtoScript M-MuLV First Strand cDNA Synthesis Kit (New England Biolabs, Ipswich, MA, USA) according to the manufacturer’s instructions with oligo dT primers. cDNA was measured by qPCR using the Luna Universal qPCR Master Mix (New England Biolabs) with a Rotor-Gene cycler (Qiagen, Germantown, MD, USA) according to the manufacturer’s instructions. Expression was calculated as the amount of cDNA from the gene of interest relative to the amount of *TEF1* cDNA in the same sample using the second-derivative maximum to determine C^T^ values.

### 2.8. Microscopy

For the localization study conducted, strains were inoculated into SDC-glucose and grown at 30 °C for 16–18 h. The overnight cultures were then diluted 1:10 into SDC-glucose, SDC-glucose-nocodazole (50 µM), and SDC-glucose-H_2_O_2_ (1.2 mM), and then incubated for 3 h at 30 °C with shaking. DNA was stained with 5 µg/mL Hoechst 15 min and resuspended in 1× PBS prior to imaging. GFP filtered sets as appropriate using constant exposure times and scaling of the strain. Cells were imaged with a Zeiss LSM confocal microscope. Images were acquired using Zeiss ZEN software. 

## 3. Results 

### 3.1. Deletion of CaMAD2 Reduces Growth

**Figure 1:**
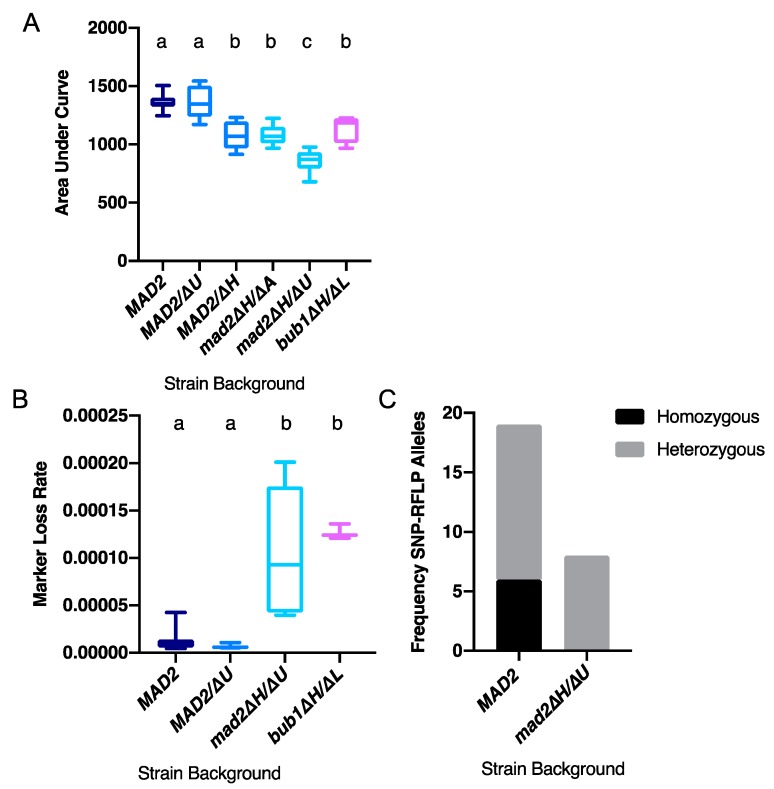
Deletion of *MAD2* reduces total growth and increases marker loss rate. (**A**) *Candida albicans* strains with/or without the deletion of *MAD2* or *BUB1* were grown in YPA-glucose at 30 °C for 24 h. Optical density (600 nm) was collected every 15 min using a Tecan incubator/plate reader. Results were calculated from the area underneath the curve of growth. Data are maximum/minimum box plots with means (indicated by the bars) calculated from at least four biological replicates, each with four cultures per condition. Significant differences are indicated with letters (ordinary one-way ANOVA, *p* < 0.0001; Tukey multiple comparison, *p* < 0.01 for letter difference). (**B**). *C. albicans* strains with/or without the deletion of *MAD2* or *BUB1* were grown overnight in YPA-glucose media, diluted with water, and plated onto YPAD for total cell counts and 5-FOA plates to select for *URA3* loss. Colony counts were used to calculate the rate of loss per cell division. Data are maximum/minimum box plots with means (indicated by the bars) calculated from at least three biological replicates, each with eight cultures per condition. Significant differences are indicated with letters (ordinary one-way ANOVA, *p* < 0.0001; Tukey multiple comparison, *p* < 0.01 for letter difference). (**C**). Alleles on distal chromosome positions in 5-FOA resistant colonies were characterized by SNP-RFLP. Each 5-FOA colony tested was characterized as homozygous for both alleles or heterozygous.

We independently constructed two different *CaMAD2* deletion strains in derivatives of *C. albicans* SC5314. In one strain, *CaMAD2* alleles were replaced by *HIS1* and *ARG4*. In the other strain, *CaMAD2* alleles were replaced by *HIS1* and *URA3*. First, we asked whether the deletion of *CaMAD2* reduced total growth and maximal growth rate in *C. albicans* by measuring optical density. Total growth was quantified by area under the curve (AUC), which varies based on lag time (when growth starts), growth rate throughout the experiment, and the carrying capacity (maximum density of the culture). Throughout, we saw a correlation between AUC and maximal growth rate, with AUC being somewhat more sensitive, as it combines several quantitative measures of growth. We found reduced growth relative to the wild-type *MAD2* control in both *mad2∆/∆* strains and the *bub1∆U/∆L* strain, as indicated by AUC (Figure 1A) and by maximal growth rates (Figure S1). Heterozygous deletion of *MAD2* has shown haploinsufficient phenotypes in other organisms [27,28]. Therefore, we also tested the growth of two independently constructed *CaMAD2* heterozygous strains with different markers used to replace one allele of *CaMAD2.* We saw moderately reduced growth as measured by AUC (Figure 1A) and maximal growth rate (Figure S1) in a *MAD2*/∆*H* strain in which one copy of *MAD2* was replaced by *HIS1* compared to the wild-type *MAD2* control. However, we did not see significantly reduced growth in a *MAD2/∆U* strain in which one copy of *MAD2* was replaced by *URA3*. One possible explanation for the difference in the two heterozygous strains is allele variation. In earlier versions of the *C. albicans* genome annotation, one allele of *CaMAD2* was thought to have a six base pair insertion and was labeled as the long-allele [19]. More recent sequencing and assembly of the *C. albicans* SC5314 genome indicated that both alleles were the same length, but had differences in the coding sequence. Therefore, although the long-allele seems to be an artifact from a sequencing error, the alleles contain non-synonymous variation in the amino acid composition and might have functional differences [29]. 

### 3.2. Deletion of CaMAD2 Increases Genome Instability

Next, we characterized the mechanisms reducing cellular fitness by measuring genome instability. Deletion of *CaMAD2* was previously shown to result in increased growth on sorbose and in homozygosis of the mating-type locus on chromosome 5, relative to strains with intact *MAD2* [19]. The sorbose selection method primarily detects chromosome 5 monosomy rather than all types of loss of heterozygosity, as sorbose resistance occurs due to reduced copy number of several genes on chromosome 5. Given the potential role of Mad2 in DNA damage responses in addition to chromosome segregation, we examined the effect of *CaMAD2* deletion using 5-FOA counter-selection against the *URA3* marker which allows detection of multiple types of loss of heterozygosity as the *URA3* gene is confined to a small region on the chromosome where it is located. Using the *mad2∆H/∆U* strain heterozygous for *URA3* on chromosome 1, we observed elevated marker loss rates per cell division (Figure 1B). We then used SNP-RFLP to determine whether 5-FOA resistance was due to smaller-scale loss of heterozygosity events, such as mutation or recombination, or whole chromosome loss resulting in whole chromosome homozygosis. For the wild-type *MAD2-*containing strain, we observed marker loss due to a mix of smaller scale events and whole chromosome loss. For the *mad2∆/∆* strain, we detected only smaller scale events as all of 5-FOA^R^ colonies tested were heterozygous at one or both chromosome ends (Figure 1C and Table S2). Therefore, the elevated *URA3* marker loss rate observed in strains lacking *MAD2* was due to elevated mutation or recombination rather than whole chromosome loss. The *MAD2/∆U* heterozygous strain did not show increased marker loss (Figure 1B), consistent with the absence of growth-related phenotypes. The *URA3* marker loss rate on chromosome 4 for *bub1∆U/∆L* was similar to the homozygous *mad2* deletion strain (Figure 1B). 

### 3.3. More Kinetochore-Microtubule Attachments Per Centromere Does Not Affect the Requirement for MAD2

The canonical role of Mad2 is to detect unattached kinetochores as part of the spindle assembly checkpoint. In *C. albicans*, each chromosome typically has one kinetochore-microtubule attachment per centromere. Overexpression of *CSE4,* encoding the centromeric histone CENP-A, results in an increased number of kinetochore-microtubule attachments per centromere. Approximately 60% of centromeres have two kinetochore-microtubule attachments when *CSE4* is overexpressed [30]. We reasoned that higher numbers of kinetochore-microtubule attachments per centromere might alter the requirement for the spindle assembly checkpoint as there are more potentially unattached kinetochores. Therefore, we next tested whether deletion of *MAD2* has synergistic phenotypes with increased numbers of kinetochore-microtubule attachments per centromere. Growth of the cells in succinate activated the *PCK1* promoter driving *CSE4*, resulting in high levels of *CSE4* expression relative to growth in glucose (repressing the *PCK1* promoter upstream of one copy of *CSE4*) (Figure S2A). We did not observe any synergistic effects of *MAD2* deletion and *CSE4* overexpression as measured by total growth (Figure S2B) or maximal growth rate (Figure S2C).

**Figure 2:**
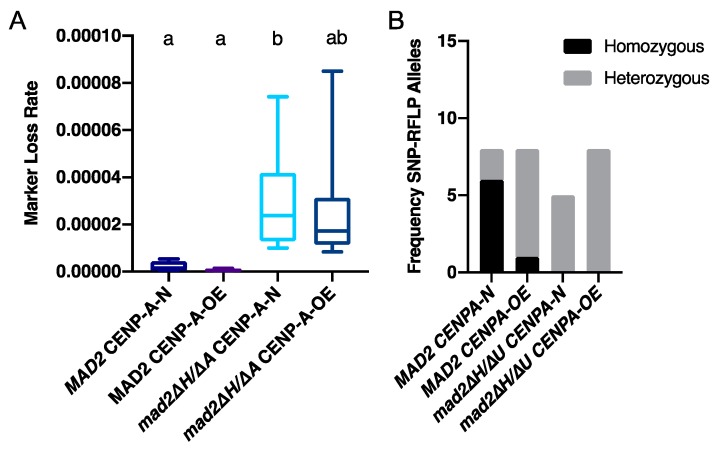
More kinetochore-microtubule attachments per centromere does not affect marker loss caused by *MAD2* deletion. (**A**). *C. albicans* strains with the conditional *PCK1* promoter regulating one copy of *CSE4* with/or without the deletion of *MAD2* were cultured in YPA-glucose (N) or YPA-succinate (OE) media. Cells were grown in each condition overnight, diluted with water, and plated onto YPAD for total cell counts and 5-FOA plates to select for *URA3* loss. Colony counts were used to calculate the rate of loss per cell division. Data are maximum/minimum box plots with means (indicated by the bars) calculated from at least three biological replicates, each with four cultures per condition. Significant differences are indicated by letters (ordinary one-way ANOVA, *p* < 0.01; Tukey’s multiple comparison test, *p* < 0.05 for letter difference). (**B**). Alleles on distal chromosome positions in 5-FOA resistant colonies were characterized by SNP-RFLP. Each 5-FOA colony tested was characterized as homozygous for both alleles or heterozygous.

Importantly, marker loss rates of a heterozygous *URA3* marker on chromosome 3 were similar in *mad2∆/∆* cells overexpressing *CSE4*, and *mad2∆/∆* cells with normal levels of Cse4 (Figure 2). Consistent with the results seen with chromosome 1, *URA3* marker loss rates on chromosome 3 were elevated in the *mad2∆/∆* strain compared to the strain with *MAD2* (Figure 2). For the strains with *MAD2*, we observed marker loss due to a mix of smaller scale events and whole chromosome loss events with and without overexpression of *CSE4*. For the *mad2∆/∆* strains with and without *CSE4* overexpression, we detected only smaller scale events, as all of the 5-FOA^R^ colonies tested were heterozygous at one or both chromosome ends (Figure 2B and Table S2). Regardless of whether *CSE4* was overexpressed, the elevated *URA3* marker loss rate in strains lacking *MAD2* was due to increased mutation or recombination rates rather than whole chromosome loss. 

### 3.4. Loss of MAD2 Increases Sensitivity to Microtubule-Destabilizing Compounds

**Figure 3:**
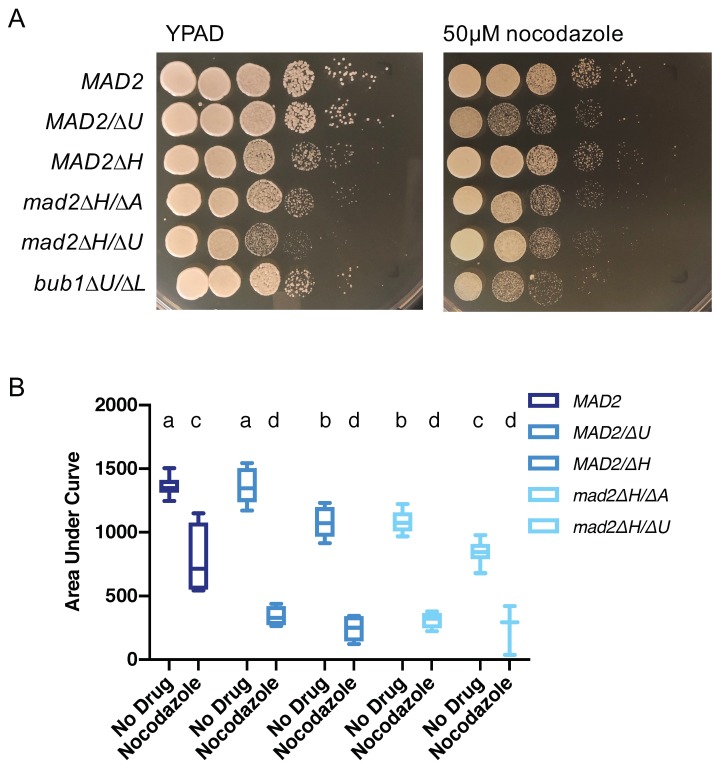
Loss of *MAD2* increases sensitivity to the microtubule-destabilizing compound nocodazole (**A**). *C. albicans* strains were spotted in 10-fold serial dilutions on YPAD or YPAD + 50 µM nocodazole plates. Relative growth was assayed after 24 h of culture at 30 °C. (**B**). *C. albicans* strains with/or without the deletion of *MAD2* were grown in YPA-glucose or YPA-glucose + 50 µM nocodazole at 30 °C for 24 h. Optical density (600 nm) was collected every 15 min using a Tecan incubator/plate reader. Results were calculated from the area underneath the curve of growth. Data are maximum/minimum box plots with means (indicated by the bars) calculated from at least three biological replicates, each with four cultures per condition. Significant differences are indicated with letters (two-way ANOVA: strain difference *p* < 0.0001, nocodazole difference *p* < 0.0001, interaction *p* < 0.0001; Tukey multiple comparison, *p* < 0.05 for letter difference).

Next, we exposed cells with and without Mad2 to treatments that induce microtubule-destabilization and DNA damage effects to further test the hypothesis that CaMad2 functions in direct spindle assembly checkpoint roles and has roles in preventing recombination and other types of sub-chromosomal genome protection mechanisms. We first confirmed the role of CaMad2 in the spindle assembly checkpoint by using nocodazole and thiabendazole. Nocodazole treatment destabilizes microtubules and increases unattached kinetochores. In several organisms, including *C. albicans*, deletion of *MAD2* increases sensitivity to nocodazole [19,31,32]. We examined growth by dilutions and spot plating on media containing nocodazole and quantifying growth in the presence of nocodazole with optical density. We observed moderately reduced growth of the strain containing *MAD2* by both measures. We also saw increased sensitivity, defined as a reduced percentage growth compared to the same strain in no drug conditions, to nocodazole for the heterozygous *MAD2/mad2∆* and *mad2∆/∆* strains with both methods of measuring growth (Figure 3). We also observed sensitivity of the *bub1∆/∆* strain on plates containing nocodazole (Figure 3A). For the AUC growth curve, the mean growth of the *MAD2* strain in nocodazole was 57% of the mean growth with no drug, while the percentages of growth in nocodazole relative to the no drug treatment were 25% and 23% for the *MAD2* heterozygous strains and 29% for the *mad2∆/∆* strains (Figure 3B). 

Thiabendazole is another drug with microtubule destabilizing mechanisms of action [33]. Thiabendazole reduced growth of the strain containing *MAD2*, and *MAD2/mad2∆* and *mad2∆/∆* strains, with the strains lacking at least one copy of *MAD2* showing increased sensitivity to thiabendazole using the total growth measurement with optical density (Figure S3). For the AUC growth curve, the mean growth of the *MAD2* strain in thiabendazole was 37% of the mean growth with no drug, while the percentages of growth in thiabendazole relative to the no drug treatment were 9% and 24% for the *mad2∆/∆* strains.

### 3.5. Deletion of MAD2 Increases Sensitivity to DNA-Damage Inducing Treatments

**Figure 4:**
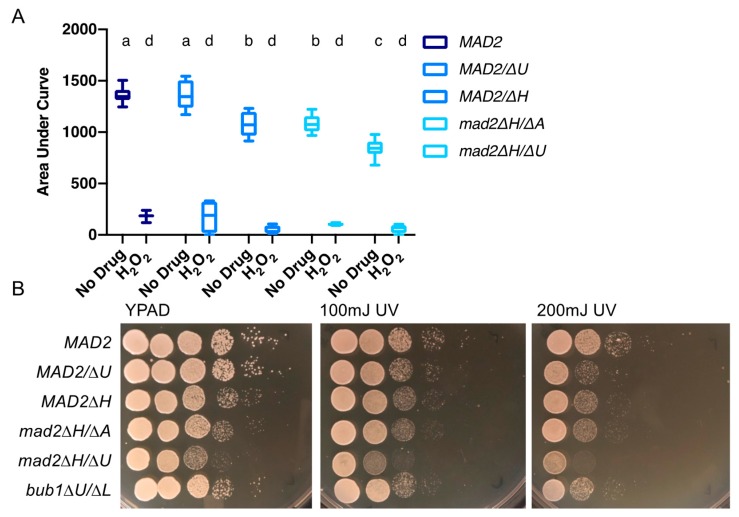
Loss of *MAD2* increases sensitivity to the DNA damage inducing agents (**A**). *C. albicans* strains with/or without the deletion of *MAD2* were grown in YPA-glucose or YPA-glucose + 1 mM hydrogen peroxide at 30 °C for 24 h. Optical density (600 nm) was collected every 15 min using a Tecan incubator/plate reader. Results were calculated from the area underneath the curve of growth. Data are maximum/minimum box plots with means (indicated by the bars) calculated from at least two biological replicates, each with four cultures per condition. Significant differences are indicated with letters (two-way ANOVA: strain difference *p* < 0.0001; hydrogen peroxide difference *p* < 0.0001; interaction difference *p* < 0.001; Tukey multiple comparison, *p* < 0.05 for letter difference). (**B**). *C. albicans* strains were spotted in 10-fold serial dilutions on YPAD. For UV treatment, plates were exposed to 100 mJ/cm^2^ or 200 mJ/cm^2^ UV light. Relative growth was assayed after 24 h of culture at 30 °C.

We grew strains with and without CaMad2 on media containing hydrogen peroxide. Hydrogen peroxide causes oxidative damage and increases DNA recombination [26]. Hydrogen peroxide (1 mM) reduced growth for the strain containing *MAD2* and for the *MAD2/mad2∆* and *mad2∆/∆* strains. The strains lacking *MAD2* showed slightly increased sensitivity to hydrogen peroxide using the total growth measurement with optical density (Figure 4A). When comparing total growth, the mean *MAD2* growth in 1mM hydrogen peroxide was 13% of the mean growth of the control, while the percentages of growth in the same concentration of hydrogen peroxide relative to the no drug treatment were 7% and 9% for the *mad2∆/∆* strains. Similarly to our results with total growth, the *mad2/∆U* strain was not sensitive to hydrogen peroxide (13%), while the *mad2/∆H* strain was sensitive (6%) (Figure 4A). We also tested the effect of UV light exposure, another condition causing DNA damage to cells. We examined growth by dilutions and spot plating followed by exposure to 100 mJ/cm^2^ or 200 mJ/cm^2^ UV light. We observed moderately reduced growth of the strain containing *MAD2* and an increased sensitivity to UV light for both *mad2∆/∆* strains (Figure 4B). 

### 3.6. CaMad2 Localizes to the Nuclear Periphery in C. albicans

**Figure 5:**
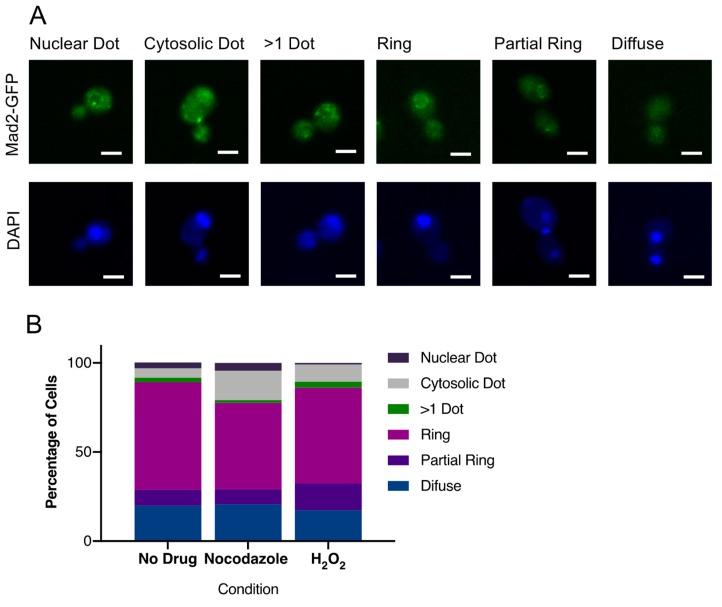
Mad2-GFP localizes to the nuclear periphery. Mad2-GFP (green, top row) expressing cells were grown in SDC with or without nocodazole or hydrogen peroxide treatment for 4 h at 30 °C and then stained with Hoechst (blue, bottom row) to allow for identification of the nuclear position within the cell. Cells were imaged by confocal microscopy at 1000× total magnification. (**A**). Mad2-GFP exhibited several localization patterns within the cells. Representative images from each phenotype are shown. Scale bars = 5 µm. (**B**). The percentage of cells with each localization pattern was quantified. Data shown are total percentages from at least 200 cells images combined from at least three separate experiments.

To determine the localization of CaMad2, we tagged the C-terminus of CaMad2 with GFP, stained the nuclei with Hoescht DNA stain, and visualized live cells by confocal microscopy. We found localization to several different patterns: one dot colocalized with the nucleus, one dot not colocalized with the nucleus, more than one dot, a complete ring surrounding the nuclear periphery, a partial ring surrounding the nuclear periphery, and a diffuse distribution of GFP in the cell (Figure 5A). A majority of the cells exhibited a ring surrounding the nuclear periphery (Figure 5B). The ring morphology was observed in both budding and non-budding cells. The most common alternative localization patterns were the formations of a Mad2-GFP cluster at a single point within the cell (dot) and diffuse localizations throughout the cell (Figure 5B). We next tested whether the localization pattern of CaMad2-GFP changed during treatment with microtubule-destabilizing or DNA damage-inducing treatments. We observed moderate (nocodazole) and slight (hydrogen peroxide) increases in the percentages of cells with dot localization patterns compared to untreated cells (Figure 5B). 

### 3.7. Increased Growth on Fluconazole Is a Shared Feature of Mutants with Altered Chromosome Segregation Checkpoint Function in C. albicans

**Figure 6:**
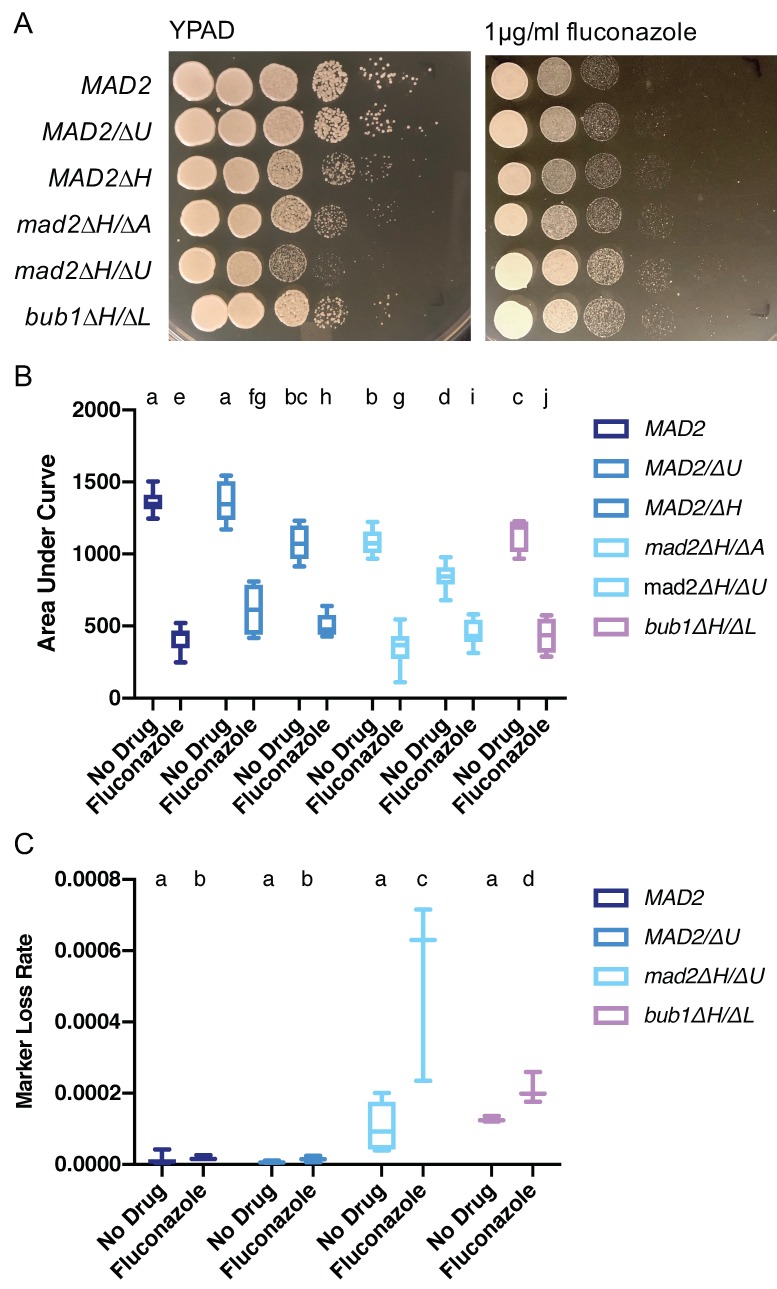
Deletion of *MAD2* increases growth on fluconazole similarly to deletion of *BUB1*. (**A**). *C. albicans* strains were spotted in 10-fold serial dilutions on YPAD or YPAD + 1 µg/mL fluconazole plates. Relative growth was assayed after 24 h of culture at 30 °C. (**B**). *C. albicans* strains with/or without the deletion of *MAD2* or *BUB1* were grown in YPA-glucose or YPA-glucose + 1 µg/mL fluconazole at 30 °C for 24 h. Optical density (600 nm) was collected every 15 min using a Tecan incubator/plate reader. Results were calculated from the area underneath the curve of growth. Data are maximum/minimum box plots with means (indicated by the bars) calculated from at least four biological replicates, each with four cultures per condition. Significant differences are indicated with letters (two-way ANOVA: strain difference *p* < 0.0001; fluconazole difference *p* < 0.0001; interaction *p* < 0.0001; Tukey multiple comparison, *p* < 0.05 for letter difference). (**C**). *C. albicans* strains with/or without the deletion of *MAD2* or *BUB1* were grown overnight in YPA-glucose media; then, diluted with water and plated onto YPAD for total cell counts and 5-FOA plates to select for *URA3* loss. Colony counts were used to calculate the rate of loss per cell division. Data are maximum/minimum box plots with means (indicated by the bars) calculated from at least six biological replicates, each with eight cultures per conditions. Significant differences are indicated with letters (two-way ANOVA: strain difference *p* < 0.0001; fluconazole difference *p* < 0.0001; interaction *p* < 0.0001; Tukey multiple comparison, *p* < 0.05 for letter difference).

*C. albicans* strains with mutations that alter chromosome segregation checkpoint function, such as *bub1∆/∆, sgo∆/∆*, and *h2a.2∆/∆* strains, have increased growth on the antifungal drug fluconazole compared to wild-type cells [17]. We hypothesized that deletion of *CaMAD2* would also alter growth on fluconazole. We examined growth by dilutions and spot plating on YPAD media containing 1 µg/mL fluconazole. We observed increased growth relative to the *MAD2* cells for both *mad2∆/∆* strains and for the *bub1∆/∆* strain, as colonies in these strains were visible in spots with higher dilutions than the wild-type strains. The increased growth was more substantial when considering the growth defects seen in these strains without drug treatment (Figure 6A). When comparing total growth in fluconazole to total growth in no drug media, fluconazole had less of an effect on growth for both *mad2∆/∆* strains and for the *bub1∆/∆* strain than for the strain with intact spindle assembly checkpoint components (Figure 6B). The heterozygous *MAD2/mad2∆* strains had an intermediate phenotype with fluconazole consistent with the haplo-insufficient phenotypes observed with other treatments as well. 

Treatment with 1 µg/mL fluconazole significantly increased *URA3* marker loss rate in the *mad2∆H/∆U* deletion strain (Figure 6C). This increase in marker loss seems to be due to an increase in whole chromosome loss, as all strains tested were homozygous in SNP-RFLP assays (Table S2). The *bub1∆/∆* strain had similar *URA3* marker loss rates to the *mad2∆/∆* strain in the absence of fluconazole, and showed an elevated loss rate with fluconazole treatment, although the magnitude of increase was less than for the *mad2∆/∆* strain (Figure 6C). We also measured the minimum inhibitory concentration (MIC) for *MAD2, mad2∆/∆,* and *bub1∆/∆* strains using a broth dilution assay in RPMI. The MIC values were ≤0.125 µg/mL for the *MAD2* strains. We observed MIC values of 0.125 and 0.25 µg/mL strains for the *mad2∆/∆* and *bub1∆/∆* strains. All of these values fall below the cutoff for fluconazole susceptibility of <8 µg/mL [34], but there was a trend towards increased MIC values in the *mad2∆/∆* and *bub1∆/∆* strains.

## 4. Discussion

In this work, we found that deletion of *CaMAD2* decreased cell growth, increased marker loss rates, increased sensitivity to microtubule-destabilizing drugs, and increased sensitivity to DNA damage-inducing treatments. We did not see an increased requirement for CaMad2 when *CSE4* was overexpressed resulting in additional kinetochore-microtubule attachments per centromere. Together, these results show that CaMad2 promotes genome stability at the chromosomal level through its canonical spindle assembly checkpoint roles and at the sub-chromosomal level through roles in preventing DNA damage and recombination. Additionally, CaMad2-GFP localized to dots, consistent with a role in kinetochore binding, and to the nuclear periphery, consistent with an additional role in DNA damage. We also found that, similarly to other strains with altered chromosome segregation checkpoint function, deletion of *MAD2* increases growth on fluconazole, and that fluconazole treatment elevates whole chromosome loss rates in the *mad2∆/∆* strain. 

A prior characterization of CaMad2 found that it is important for accurate chromosome segregation, survival in high levels of hydrogen peroxide, and virulence in a mouse model of infection [19]. We found that deletion of *MAD2* in *C. albicans* results in a slow growth phenotype. This is similar to previous observations that *mad2∆* strains of the Fusarium head blight fungus *Fusarium graminearum* and the multinucleate fungus *Ashbya gossypii* had reduced growth and viability, likely due to high levels of aneuploidy [35,36]. Interestingly, haploinsufficiency was also observed in our experiments with *C. albicans* and in *A. gossypii* [35]. *MAD2* haploinsufficiency also causes chromosome instability in human cancer cells and mouse embryonic fibroblasts [37], while overexpression of the *MAD2* gene is also associated with tumor formation and poor cancer prognosis [38,39]. The phenotypes observed with both decreased and increased levels of *MAD2* suggest that MAD2’s function relies on a particular level of expression in the cell. This may be related to the importance of the protein’s stoichiometry. Mad2 protein is found in both open and closed conformations, which dimerize to each other. This dimerization helps convert the open form into the closed form that then binds to Cdc20 to form the MCC [40]. Thus, up or down regulation of the protein may affect the stoichiometry and the amount of bound closed Mad2, which could disrupt the spindle assembly checkpoint.

Previously, Bai et al. showed elevated sorbose resistance rates and homozygosis of the mating type locus in strains lacking *CaMAD2* suggesting that CaMad2 has roles in chromosome segregation accuracy in *C. albicans* [19]. The sorbose resistance assay measures monosomy of chromosome 5 due to the presence of multiple sorbose utilization genes on chromosome 5. We used selection of 5-FOA resistant colonies to measure loss of the *URA3* marker gene. Since this assay requires loss of only a single gene, it can detect multiple types of loss of heterozygosity events. Interestingly, in strains lacking *CaMAD2*, we saw elevated *URA3* marker loss rates. These elevated loss rates in rich media were primarily due to smaller-scale events, such as mutation and recombination, as all colonies were still heterozygous. Overall, these results show that deletion of *CaMAD2* causes both chromosome loss phenotypes and elevated small-scale DNA changes. 

Our treatment sensitivity assays also support a model where CaMad2 has roles in chromosome segregation and preventing DNA damage. We confirmed the sensitivity of strains lacking CaMad2 to 50 µM nocodazole, a microtubule destabilizing drug, in our experiments. Bai et al. showed reduced viability of strains lacking CaMad2 following short-term exposure to high concentrations (147, 88, and 33 mM) of hydrogen peroxide [19]. We tested longer-term exposures (24 h) to a lower concentration (1 mM) and also found sensitivity of strains lacking CaMad2 to this lower concentration, as demonstrated by reduced growth. The hydrogen peroxide results, together with the ultraviolet light sensitivity and increased *URA3* marker loss due to recombination or other smaller scale events observed with the *Camad2∆/∆* strain, demonstrate that CaMad2 also has a role in preventing DNA damage. Throughout our work, we saw similar phenotypes with *Camad2* deletion and *Cabub1* deletion strains, and in a few experiments, even saw stronger phenotypes with the *mad2* deletion strain. Prior work in budding yeast and fission yeast found that *bub1* mutants had more severe chromosome segregation defects than deletions of mitotic checkpoint components, such as Mad1, Mad2, and Mad3 [41]. There are several possibilities to explain why the phenotypes were more similar in *C. albicans*, including the presence of an H2A form that cannot be phosphorylated by Bub1 [17] and/or the important role of CaMad2 in DNA damage responses.

Additionally, prior work has shown that the nuclear periphery localization pattern of Mad2 is associated with the response to DNA damage [6]. The nuclear periphery localization pattern we observed with CaMad2 is consistent with the hypersensitivity to DNA damage and the high proportion of sub-chromosomal marker loss events. However, CaMad2 localization to the nuclear periphery appears to be the default location rather than a specific response to DNA damage, as the treatment with hydrogen peroxide did not increase the proportion of cells with a complete ring of CaMad2-GFP at the nuclear periphery. These results are also consistent with the result in *Saccharomyces cerevisiae* that Mad1 and Mad2 only bind to kinetochores when there is a problem with the spindle or kinetochore [42]. Together, these results indicate that the DNA damage response through Mad2 is conserved in *C. albicans*. 

There are a few different proposed mechanisms in *S. cerevisiae* for the role of Mad2 in preventing DNA damage. Mad2 prolongs cell cycle arrest in cells with unrepaired double-strand breaks, suggesting that it may be important for allowing cells time to repair the DNA damage [43]. In *S. cerevisiae*, Mad2 also potentially has roles in nucleotide excision repair pathways, as *mad2∆* shows synthetic lethality with the DNA damage response genes *RAD10* and *RAD23*, which are subunits of nucleotide excision repair factor 1 and nucleotide excision repair factor 2, respectively [44]. Additionally, recent evidence shows that Mad2 promotes translation of the S-phase cyclins Clb5/6 and contributes to cell survival in response to replication stress when Rad53 is not functional [7]. In the future, it will be interesting to further characterize the mechanisms by which CaMad2 contributes to DNA damage responses in *C. albicans*. 

It will also be critical to further explore the interactions between spindle assembly checkpoints and the development of drug resistance. Deletion of *MAD2* increases sensitivity to antifungals that target microtubules, such as thiabendazole in *C. albicans* and *F. graminearum* [36], but deletion of *CaMAD2* increases growth on the antifungal fluconazole. This observation is similar to other strains with altered chromosome segregation checkpoint function, including *bub1∆/∆, sgo∆/∆,* and *h2a.2∆/∆* strains [17], suggesting that disruption of any of these genes may promote the evolution of drug resistance in *C. albicans*. Cells lacking *CaMAD2* had slightly elevated MIC values relative to cells without disruption of *CaMAD2*, but both strains were still below the MIC cutoff for resistance. It would be interesting to test whether cells lacking *CaMAD2* evolve fluconazole resistance differently than cells with functional *CaMAD2* following repeated exposure to fluconazole. 

Fluconazole resistance is associated with elevated levels of aneuploidy. In one set of clinical and laboratory strains, approximately 50% of fluconazole resistant strains contained a whole chromosome or segmental aneuploidy, while 10% of fluconazole sensitive strains were aneuploid [45]. We found that the elevated marker loss rate seen in rich media conditions with the *mad2∆/∆* strain increases further upon exposure to treatment. Importantly, the type of marker loss events also changed from sub-chromosomal events to whole chromosome homozygosis, indicative of whole chromosome aneuploidy. Exposure to fluconazole induces aneuploidy through the separation of the nuclear membrane segregation and cytokinesis processes [46]. Our results show that the absence of *CaMAD2* may potentiate fluconazole-induced aneuploidy, which leads to better growth on low levels of fluconazole for *mad2∆/∆* strains. 

## 5. Conclusions

In this work, we found that CaMad2 promotes genome stability through its direct spindle assembly checkpoint role in promoting accurate chromosome segregation and through reducing smaller scale chromosome changes due to recombination and DNA damage repair. Furthermore, the frequent nuclear periphery localization pattern of CaMad2-GFP is consistent with a role in DNA damage response as similar patterns have been seen in other organisms. Together, our results demonstrate that a Mad2-dependent DNA damage response is conserved in *C. albicans*. We also found that deletion of *MAD2* increases growth on fluconazole similar to other strains with altered chromosome segregation checkpoint function. These results indicate that disruption of spindle assembly checkpoint genes, including *MAD2*, may promote the evolution of azole drug resistance in *C. albicans*. 

## Supplementary Materials

The following are available online at https://www.mdpi.com/2073-4425/10/12/1013/s1. Figure S1: Deletion of *MAD2* decreases growth rate, Figure S2: Growth of strains with and without MAD2 are not affected by increased kinetochore-microtubule attachments per centromere, Figure S3: Loss of *MAD2* increases sensitivity to the microtubule-destabilizing compound thiabendazole, Table S1: *C. albicans* strains used in this study, Table S2: Number of heterozygous and homozygous *URA3* marker loss events measured by SNP-RFLP, File S1: Growth Curve Macros Excel Sheet Template.
