# Supplementary material for: CaMad2 Promotes Multiple Aspects of Genome Stability Beyond Its Direct Function in Chromosome Segregation
Source: Genes (Basel). 2019 Dec 5;10(12):1013. doi: 10.3390/genes10121013 (PMC6947305; doi:10.3390/genes10121013)
Supplement: Supplementary file 1 [file genes-10-01013-s001.zip › SupplementalMaterial/BurrackSupplementalTables.docx]

**Supplemental Table S1.** *C. albicans* strains used in this study

Strain # Strain Details Reference

LB2 *ura3Δ::λimm434/ura3Δ::λimm434 INT1/int1::URA3* (Forche et al., 2011)

LB11 *ura3Δ::λimm434/ura3Δ::λimm434 his1::hisG/his1::hisG* (Burrack et al., 2011)

*arg4::hisG/arg4::hisG cse4::PCK1pr-CSE4(URA3)/CSE4*

LB112 *ura3Δ::λimm434/ura3Δ::λimm434 his1::hisG/his1::hisG* This work

*arg4::hisG/arg4::hisG mad2::ARG4/mad2::HIS1*

LB115 *ura3Δ::λimm434/ura3Δ::λimm434 his1::hisG/his1::hisG* This work

*arg4::hisG/arg4::hisG mad2::ARG4/mad2::HIS1*

*cse4::PCK1pr-CSE4(URA3)/CSE4*

LB120 *ura3Δ::λimm434/ura3Δ::λimm434 his1::hisG/his1::hisG* This work

*arg4::hisG/arg4::hisG mad2::HIS1/mad2::URA3*

LB121 *ura3/ura3 his1::hisG/his1::hisG MAD2/mad2::URA3* This work

LB125 *leu2Δ/leu2Δ, his1Δ/his1Δ, arg4Δ/arg4Δ, ura3Δ/URA3,* (Brimacombe et al., 2019)

*iro1Δ/IRO1, bub1Δ::C.m.LEU2/bub1Δ::C.d.HIS1*

LB127 *leu2Δ/leu2Δ, his1Δ/his1Δ, arg4Δ/arg4Δ, ura3Δ/URA3,* (Brimacombe et al., 2019)

*iro1Δ/IRO1, BUB1/bub1Δ::C.d.HIS1*

LB129 *ura3Δ::λimm434/ura3Δ::λimm434 his1::hisG/his1::hisG* This work

*arg4::hisG/arg4::hisG MAD2/mad2::HIS1*

LB130 *ura3Δ::λimm434/ura3Δ::λimm434 his1::hisG/his1::hisG* This work

*arg4::hisG/arg4::hisG cse4::PCK1pr-CSE4(URA3)/CSE4*

*MAD2/MAD2-GFP-HIS1*

**Supplemental Table S2.** Number of heterozygous and homozygous *URA3* marker loss events measured by SNP-RFLP

|  | **Homozygous** | **Heterozygous** |
| --- | --- | --- |
| *MAD2* | 6 | 13 |
| *mad2ΔH/ΔU* | 0 | 8 |
| *MAD2 CENPA-N* | 6 | 2 |
| *MAD2 CENP-A-OE* | 1 | 7 |
| *mad2ΔH/ΔA CENP-A-N* | 0 | 5 |
| *mad2ΔH/ΔA CENP-A-OE* | 0 | 8 |
| *mad2ΔH/ΔU* + 1µg/ml fluconazole | 8 | 0 |
