# Supplementary material for: CaMad2 Promotes Multiple Aspects of Genome Stability Beyond Its Direct Function in Chromosome Segregation
Source: Genes (Basel). 2019 Dec 5;10(12):1013. doi: 10.3390/genes10121013 (PMC6947305; doi:10.3390/genes10121013)
Supplement: Supplementary file 1 [file genes-10-01013-s001.zip › SupplementalMaterial/SupplementalFigures.pdf]

Figure S1

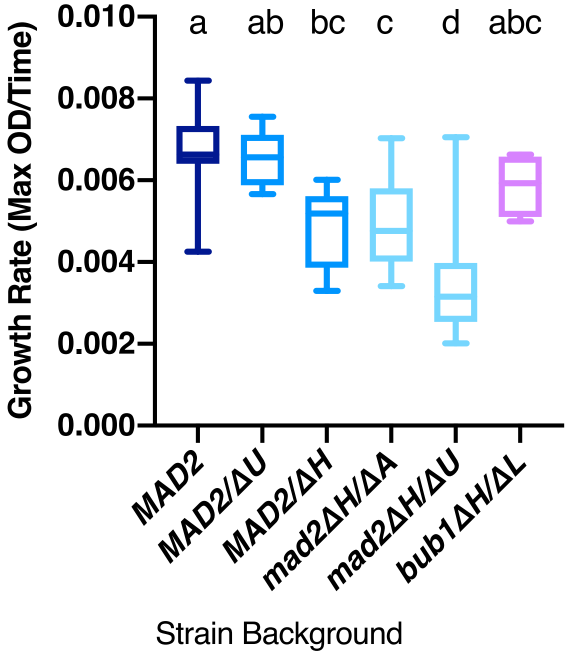

Supplemental Figure S1: Deletion of *MAD2* decreases growth rate. C. albicans strains with/or without the deletion of *MAD2* or *BUB1* were grown in YPA-Glucose at 30ºC for 24 h. Optical density (600nm) was collected every 15 min using a Tecan incubator/plate reader. Results are the maximum growth rate during the time period. Data are max/min box plots with means (indicated by the bars) calculated from at least four biological replicates, each with four cultures per condition. Significant differences are indicated by letters (Ordinary one-way ANOVA, $p<0.0001$; Tukey multiple comparison, $p<0.05$ for letter difference).

Figure S2

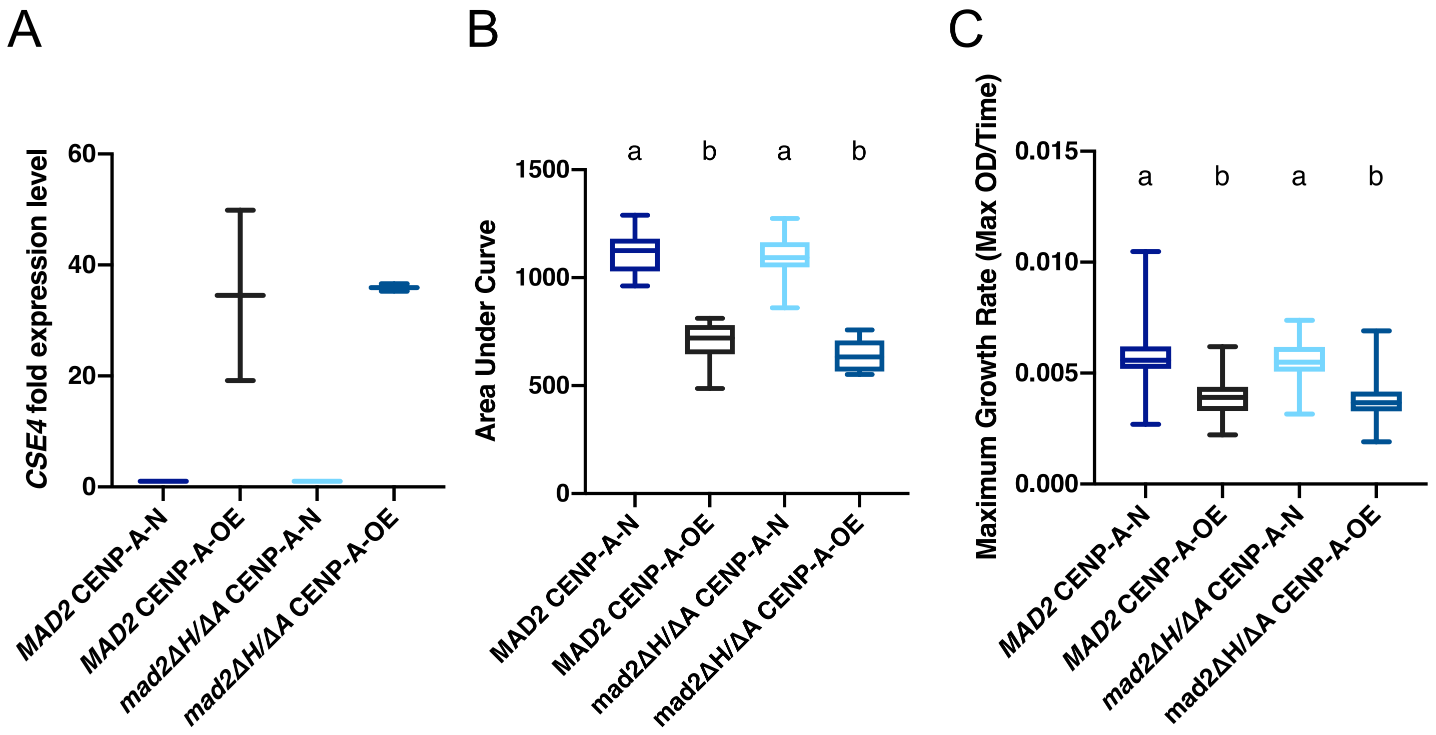

Supplemental Figure S2: Growth of strains with and without *MAD2* are not affected by increased kinetochore-microtubule attachments per centromere. A) C. albicans strains with the conditional *PCK1* promoter regulating one copy of *CSE4* with/or without the deletion of *MAD2* were cultured in YPA-Glucose (N) or YPA-Succinate (OE) media at 30°C for 4 h. mRNA levels of *CSE4* relative to the reference gene *TEF1* were measured by qRT-PCR. B) *C. albicans* strains with the conditional *PCK1* promoter regulating one copy of *CSE4* with/or without the deletion of *MAD2* were cultured in YPA-Glucose (N) or YPA-Succinate (OE) media at 30°C for 24 h. Optical density (600nm) was collected every 15 min using a Tecan incubator/plate reader. Results are calculated from the area underneath the curve of growth. Data are max/min box plots with means (indicated by the bars) calculated from at least thirteen experiments, each with four cultures per condition. Significant differences are indicated by letters (Tukey's multiple comparison test, $p < 0.0001$). C) Results are the maximum growth rate during the time period. Means (indicated by the bars) are from the growth rates calculated from at least thirty experiments, each with four cultures per condition. Significant differences are indicated by letters (Tukey's multiple comparisons test, $p < 0.0001$ for letter difference).

Figure S3

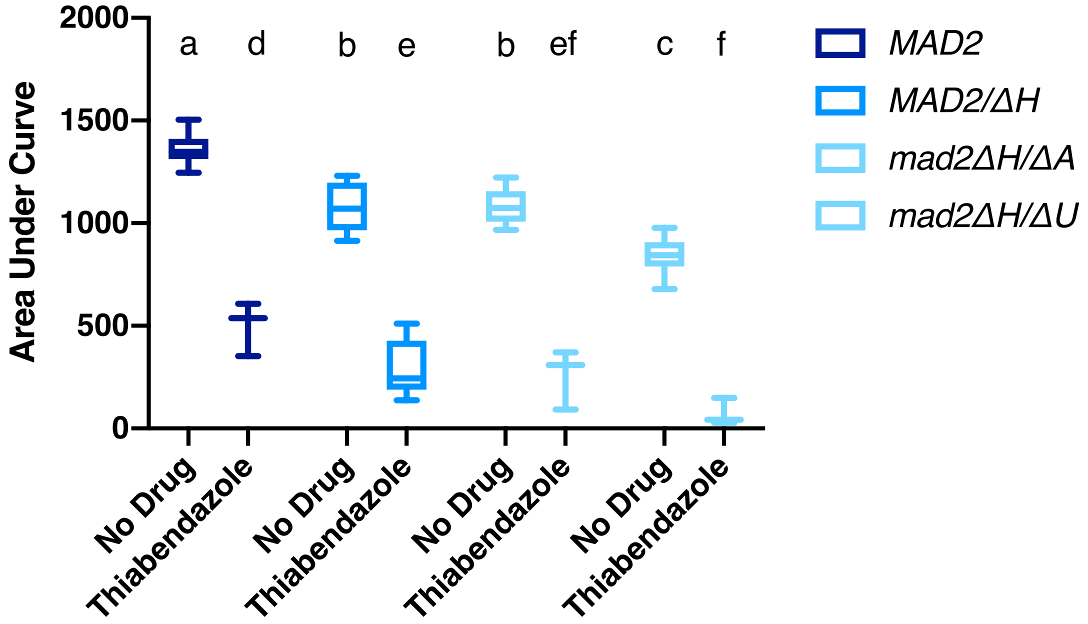

Supplemental Figure S3: Loss of *MAD2* increases sensitivity to the microtubule-destabilizing compound thiabendazole *C. albicans* strains with/or without the deletion of *MAD2* were grown in YPA-Glucose or YPA-Glucose + 222µg/mL thiabendazole at 30°C for 24 h. Optical density (600nm) was collected every 15 min using a Tecan incubator/plate reader. Results are calculated from the area underneath the curve of growth. Data are max/min box plots with means (indicated by the bars) calculated from at least three biological replicates, each with four cultures per condition. Significant differences are indicated with letters (Two-way ANOVA: Strain difference $p<0.0001$, thiabendazole difference $p<0.0001$); Tukey multiple comparison, $p<0.05$ for letter difference).
